# The Status of Mechanical Vibration in Therapeutics

**Published:** 1904-12

**Authors:** 


### THE STATUS OF MECHANICAL VIBRATION IN THERAPEUTICS.

Mechanical vibration, which properly includes two forms of application—spinal stimulation and vibra-massage—has established an important place in therapeutics, which it is certain to fill to the advantage of suffering humanity. That it can, however, fill the place which the narrow osteopathic notion would give it in therapeutics, is too absurd to contemplate, and those who would underrate the value of other physical therapeutic measures in comparison with mechanical vibration, belong to the school of thought in which small minds, confining themselves to small circles, fail to comprehend the great scope of a comprehensive use of different measures to meet different indications. If comparisons were to be drawn, mechanical vibration, while filling a valued field, could never cope in its extent of application with the use of the different electrical modalities; for the surgical procedures of the continuous current, the employment of the electrical current in the production of light and the X-ray, and the uses of high potential electricity, cover the widest range in the field of therapeutics to-day.

The electro-therapeutist, however, if he chooses to be designated by the title, is very narrow-minded if he will not embrace the application of mechanical vibration as an adjunct in the treatment of his patients. What we look for to-day, is not a controversy as to the relative merits of therapeutic agents, but a broader conception and selected discrimination for the application of the different measures and methods as shall best suit conditions as they arise. It is to be deprecated that there are any so narrow and commercial in their views as to set forth a narrow doctrine, which would exclude all but one of the valuable physical therapeutical measures.—*Journal Advanced Therapeutics.*

